# Perception of Iranian Middle-Aged Women Regarding Moral Health Concept: A Content Analysis

**DOI:** 10.5539/gjhs.v7n3p267

**Published:** 2014-11-30

**Authors:** Mahvash Salsali, Nasrin Rezaee, Naimeh Seyedfatemi, Zahra Rahnavard

**Affiliations:** 1 School of Nursing and Midwifery, Tehran University of Medical Sciences, Tehran, Iran; 2 School of Nursing and Midwifery, Zahedan University of Medical Sciences, Zahedan, Iran; 3 Center for Nursing Care Research, School of Nursing and Midwifery, Iran University of Medical Sciences, Tehran, Iran

**Keywords:** moral health, middle-aged women, qualitative study

## Abstract

**Introduction:**

The present study aimed at exploring and describing the perception of moral health from middle-aged women standpoints. Women’s decisive role in family is undeniable. In the family which is built upon tradition, faith and ethics, this is women’s principle which is represented in the moral health of the individual and the society, deals with the nature of the vice and virtue. This study attempted to identify the perception of Iranian middle-aged women about the concept of moral health.

**Method:**

The present study completed through a content analysis method. Twenty two middle-aged women were recruited through purposive sampling. Data were granted by face to face, semi-structured interview.

**Result:**

Our major categories are devotion, preserving moral values and moral challenges. Devotion category includes subcategories such as prioritizing the health of family members and trying to save marriage. Preserving moral values category includes subcategories such as respecting values and consolidating beliefs over time. Moral challenges category consists of individual and familial challenges subcategories.

**Conclusion:**

Moral health is of high importance which affects various dimensions of individual, social and familial life. The findings of the present study presented new dimensions of middle-aged women’s health regarding moral health which can finally have different consequences on familial and social moral health.

## 1. Introduction

Women’s’ health is a broad concept and bears various dimensions like physical, mental and moral health affected by various factors such as individual, social and familial ones (Ahmadi et al., 2014; Leipert & Reutter, 2005). Up to now, physical health, which is resulted from individual factors like biological differences concerning woman’ s gender, and her different needs have been considered in many studies (Ahmadi et al., 2014; García-CalventeMdel et al., 2012; Vedadhir, Sadati, & Ahmadi, 2008). It seems that the effect of gender is evident in all dimensions of women’s physical health (Ahmadi et al., 2014; Parvizy, Kiani, & Ivbijaro, 2013), but it must be noted that woman’s health is not merely dependent upon gender. It is rather affected by various factors such as functions she is responsible for. Here health, consequently, can affect the health of others (Ahmadi, Farzadi, & Alimohammadian, 2012). Family is of those institutions rooted in the traditions, beliefs and moralities. In fact, individual’s morality is formed in the family, and consequently transferring these beliefs to the society is feasible thorough family. This silent responsibility is put upon woman (Pour Esmaeili & Mozaffari, 2011; Momin, Chung, & Olson, 2014).

But based on the definition of health, offered by the World Health Organization, which is welfare state of complete physical, mental and social, health and the absence of disease or disability, it seems that this dimension of health-moral health- is less concentrated. Reviewing studies show that woman’s physical, mental and social health has been so far taken into consideration in different perspectives (Parvizy, Kiani, & Ivbijaro, 2013; Colbert, Kim, Sereik, & Erlen, 2010; Fasihi Harandy et al., 2012; Parvizy, Naseri, Seyed Fatemi, & Ghasem Zadehkakroudi, 2010). Viewing ethics from this perspective, which talks about the nature of vice and virtue (Pour Esmaeili & Mozaffari, 2011), shows that moral issues can affect individual and social health. As the finding of a study showed, women’s health is in sharp contrast with their roles as mothers and wives which presented moral issues (Alyaemni, Theobald, Faragher, Jehan, & Tolhurst, 2013). However, it seems moral issues are faded in studies concerning middle-aged women’s health. Midlife with its consequences is faced with changes and many moral attitudes which can affect woman’s various health aspects. One of the consequences can be self-neglecting which affects not only woman’s health but also the health of the family (Sharifi, Anoosheh, Foroughan, & Kazemnejad, 2014; Chiang et al., 2012).

Due to the serious effect of moral issues on individual, family and social health, it seems considering and paying attention to the experience of middle-aged woman is one of the best ways to attain information about moral health because in this age not only do they care about their health but, due to their fundamental role in family, they can also affect the family’s and society’s moral health. Due to the sophistication of the middle-aged woman, their effectiveness on moral issues and the reflection of moral issues on the health of individuals, family and society, doing this research in keeping with the moral health from the perspective of middle- aged woman is essential. We, therefore, decided to perform a qualitative study in this regard because we found out in reviewing literature that there is lack of information, especially of qualitative studies can prove helpful in explaining woman’s viewpoints using their expressions about moral health. Using qualitative approach in the present study can help the researcher enter into middle-aged woman’s perceptual world. The purpose of this study is to explain middle-aged women’s perception about moral health concept.

## 2. Method

The present study is a qualitative one done using content analysis method. This method is one of the qualitative approaches and is also one of the qualitative data analysis methods. Content analysis in which raw data are summarized based on deduction and categorized is the method of analyzing written, oral or visual messages. Conventional content analysis categories and their names are rooted in data content (Elo & Kyngas, 2008).

### 2.1 Participants and Data Collection

In this study, participants are chosen by purposive sampling method. Sampling among middle-aged women is done with the maximum variety such as educational level, marital and occupational status to attain data saturation. This study is done with 22 middle-aged women in Zahedan, Iran. Midlife (40–60) and not suffering from server physical and mental disease were criteria to choose participants. The place to interview was natural environment which made accessibility to women feasible and was appropriate to qualitative research method. In so doing, interviews were done in places such as homes or work places. Semi-structured face to face interview was used to gather data. At first, a general question was presented, “What is your idea about moral health?” To attain more information, interview continued asking following questions such as “What is your idea in this regard?” or “explain more, please”. The time of interview was between 45 to 60 minutes. With their informed consent, the interviews were digitally recorded. Each participant was once interviewed. Therefore, 22 interviewed were done.

### 2.2 Data Analysis

Data analysis was simultaneously performed with data gathering. Interviews implemented text, after several reviews, was broken down into constituent semantic units and the smallest semantic units. Then, codes were reviewed to be classified in categories and subcategories based on their semantic similarities. To ensure the accuracy of data, different methods were applied throughout the study. Participants’ reviewing was used, and the codes not mentioning their viewpoints were modified. Two faculty members of nursing who mastered qualitative study and were specialist in woman’s health scope reviewed the interview text, codes and categories. Allocating sufficient time to gather and analyze data, immediate implementing data, and simultaneous data analyzing allowed the acceptance of the data.

### 2.3 Ethical Consideration

Ethical compliance by the researchers in the study necessitated obtaining permission to conduct the research ethics committee of Tehran University of Medical Sciences, informed consent from study participants after presenting the aims of the study, allowing participants to withdraw from the partnership at any time, maintaining anonymity and confidentiality of the participants’ information and participants’ possession of information on request.

## 3. Results

Participants’ deep description identified three categories: devotion, preserving morality and moral challenges (Table 1).

**Table 1:** Categories and subcategories

Categories	Subcategories
**Devotion**	Prioritizing family member’s health Trying to save marriage
**Preserving morality**	Respecting values Consolidating beliefs over time
**Moral challenges**	Family challenges Individual challenges

### 3.1 Devotion

This category emphasized on women’s devotional aspects which prioritized family members’ health and tried to save marriage.

#### 3.1.1 Prioritizing Family Member’s Health

Middle-aged women feel high responsibility for the members of the family. It was so deep that they postponed their related health issues. One of the women said, *“If I cared about myself, I could have my own medical test done when I took my brother from one doctor to another. Two months ago, I had one to do, but I hadn’t time to have the blood and urine test. I have smear test to be readied for surgery, but I’ve not done that yet. I’m busy with my brother’s disease.”* (Participant 5).

Another participant said, *“My sister was a premature baby, and I was pregnant, but nobody knew that since my kids were grown up and I was rather old, I was shy to say I was pregnant. I was so engaged with my sister that I was aborted my child. I feel devoted to my family, and I’ll do my best for them.”* (P. 8).

#### 3.1.2 Trying to Save Marriage

A middle-aged woman devotes herself thoroughly to save marriage. A participant in this regard said, *“At my age, I see everything of life inside the woman. If I can well educate my daughter, she can save a family under any circumstances because a woman can sacrifice herself in hard times.”* (P. 11).

### 3.2 Preserving Morality

This category emphasized women’s moral beliefs in respecting values and preserving beliefs over time.

#### 3.2.1 Respecting Values

This subcategory showed that middle-aged women try to preserve moral values and beliefs at home and in the society. About respecting family values, a participant said, *“On some occasions, I drew in my horn. I found out it doesn’t worth to disrespect my husband. If you’re pertinacious, men get worse, but If you’re calm and respect them, they’ll respect you more. I came to this conclusion if I respect my husband, he will respect me, too.”* (P. 19).

Concerning respecting the values of society, another participant said, *“I learned everything from schoolmasters and teachers. They really taught us life lessons, and their personality was an index for us to learn from them. Therefore, I respected them, but nowadays youngsters learn nothing and claim too much, respect nobody. I don’t accept that they say there’s no job, and it is difficult to have an independent life. One the other hand, I believe we raised our children lazy. This is us who play the major role in children’s respecting social values.”* (P. 20).

#### 3.2.2 Consolidating Beliefs Over Time

Based on the viewpoint of the middle-aged women, their beliefs get consolidated during this phase of life. One of the women pointed out, *“One’s expectancy level should grow appropriate to life. One must over time come to this conclusion that she must do her best and go forward step by step. Everyone wants to build a good life just in few days. For example, mothers give dowry to their daughters on marriage time. This prevents newly married couple from trying to get whatever they need. But this belief for us who tried to equip our home appliances gradually was enjoyable. Now, I try gradually to obtain whatever I need and try to teach it to my kids.”* (P. 6).

Women also asserted that they are faithful to the governing social traditions under any circumstances in their lifetime. Here is a sample of such ideas: *“Now, I’m very impatient. I can’t tolerate guests any more, but I still tolerate if I have guests at home because I believe guests are God’s friends (a Persian proverb). It never happened that we have no guests. A guest or two is something we always have at our home.”* (P. 17).

### 3.3 Moral Challenges

This category covers individual or familial moral challenges that middle-aged women face and their loyalty to moral issues which are against their will.

#### 3.3.1 Familial Challenges

One of the challenges for women was changes in their functions and accepting new ones which affected the structure of their family life. One of the participants said,

*“The gap between my husband and I gets bigger because of the presence of the others. I’m more engaged with my grandchildren and spend most of my time with them. My husband envies, but I try to do what pleases him. Of course, I myself think there’s a gap between us, and I pay less attention to him. I don’t know what to do.”* (P. 11).

Another participant faced family challenges rooted in fatigue about the present situation. She expressed her distress as following, *“Last night, I cried and shouted in the alley because my son is addicted. My mom came and asked the reason. I said I’m sick of this kid. He asks me for money. If I don’t, he threatens me to bleed himself to death. Because I fear he kills himself, I have to give him the money even if it is the home rent. I’m tired of him.”* (P. 12).

#### 3.3.2 Individual Challenges

Women believe they are confronted with some moral issues in their lifetime which they have to accept them, although not agreeing with them. These issues show their adverse effects in women’s lives over the time. One of the participants mentioned,

*“The patience I’ve had so for impresses me. Maybe, I was younger then and could easily tolerate, but now I can’t. It comes a time you can’t tolerate any more. Now, I’m more meticulous about events, while I cared less when I was younger. Now I think I’m not as patient as I used to be before, and this affected me and changed my behavior. I’m out of patient now.”* (P.10)

Another participant pointed out that paying attention to moral issues made her accept some situations, *“Now, if I’m decided to remarry, what will happen to my kids? No man accepts to raise the kids of another man. What can I do with these two kids? I can’t do anything, I can’t make any decisions, and I have to get along with the situation.”* (P.15)

## 4. Discussion

In this study, middle-aged women’s perception about moral health was explained. The findings showed middle-aged women’s moral health is dependent upon concepts such as devotion, preserving beliefs and moral challenges. Participants believed devotion to be presented through items such as prioritizing family member’s health and trying to maintain a lifestyle. It seems devotion plays a pivotal role in women’s moral health. Since health, in its general sense of the word, is paying attention to and caring about yourself, but the participants’ statements from the perspectives of women’s moral health showed that moral health is equal to neglecting yourself and paying attention to others. Therefore, a category named devotion was created. In other word, women’s moral health is just meaningful when caring about other members of family is prioritized. It can also be inferred that devotion in middle-aged women is an emblem of presenting them as the cornerstone of the family. In so doing, the findings of a qualitative study showed devotion as a burdensome effort. In this study, participants pointed out that women suffer from burdensome effort for the family and compared them as millstones (Parvizy, Naseri, Seyed Fatemi, & Ghasem Zadehkakroudi, 2010). The results of another qualitative study showed that women found themselves responsible for the health of the children and the family. They, as a result, do not hesitate from any sacrifice and selflessness (Parvizy, Seyyed Ftemi, & Kiani, 2008).

The findings of the present study were consistent with the findings of other studies performed in Iran’s cultural context, and it was evident in them all that mothers, relying on their own maternal roles, sacrifice themselves for their children (Parvizy, Naseri, Seyed Fatemi, & Ghasem Zadehkakroudi, 2010; Parvizy, Seyyed Ftemi, & Kiani, 2008). However, in a study conducted in Saudi Arabia, the result showed that women are dissatisfied with their roles as mothers and wives and found it in contrast with their health (Alyaemni, Theobald, Faragher, Jehan, & Tolhurst, 2013). The following must be acknowledged that women’s devotion and sacrifice covers a broad range in our study; that is, not only do women devote themselves to their children, but they also do the same for other members of the family such as sisters, brothers, mothers and fathers. In this regard, the results of a study conducted in Canada’s cultural context showed that women such abilities are rooted in social and cultural issues of the society (Leipert & Reutter, 2005). In the researcher’s viewpoint, devotion to all members of the family is identifiable because participants in the present study live in a culture in which adherence to life roots as broad families is evident. In fact, participants’ statements emphasized that woman is the cornerstone of the family and has the power of devotion. This ability is related not only to her maternal role but also to her gender. Women’s moral health, therefore, is made out of their abilities. Based on the findings of the present study, middle-aged women’s moral health was to respect the values and consolidate the beliefs. The findings of our study emphasized that health means life values and values which connect individuals to the world. The present study showed whenever middle-aged women respect family members, they are mutually respected, too. Whenever they are visited by family members, they feel calm. It is inferred when children come to visit their parents, they give them a sense of wellness. On the other hand, our findings were consistent with the results of a study conducted in Northern Canada in which native women believe their regional cultural beliefs to be involved in their health (Parvizy, Kiani, & Ivbijaro, 2013).

To consolidate the beliefs, the findings of our research showed that women found out over time that life is to be made gradually and step by step. By showing gradual and progressive effort or in other words, by presenting life mobility, they did their best to transfer respecting social values to the next generation. Findings showed women’s moral health happens in the light of preserving values and consolidating beliefs. These results were consistent with the findings of a research declaring women are growers of human beings, and women as growers of other people can be effective in preserving and consolidating morality in society (Parvizy, Seyyed Ftemi, & Kiani, 2008). In transferring beliefs, findings of another study showed that Indian women who long immigrated to the U.S transferred their beliefs to their children (Momin, Chung, & Olson, 2014).

In addition, the results of another research showed that maintaining cultural beliefs plays a vital role in regional women’s health that had no way out but accepting cultural changes (Leipert & Reutter, 2005). Meanwhile, the results of another study showed that the Mexican families after they immigrated to the U.S, faced challenges transferring their beliefs to their children (Turner, Navuluri, Winkler, Vale, & Finley, 2014).

Reviewing researches indicated that preserving morality happens in the light of their respecting and adherence. What is certain is that transferring these beliefs plays a vital role in the health of the family and the society. It is just possible through women. As a default, women in other studies performed are the cornerstone of the health of the family and the society (Colbert, Kim, Sereik, & Erlen, 2010).

Another finding of the present study was moral challenges overshadowing women’s adherence to moral concepts. Among family challenges which middle-aged women are confronted with are new functions resulted from getting older or the marriage of the children. Of these functions is the grandmother role. Following this function, other responsibilities such as helping children in caring of the grandchildren and children’s commuting can be added. This new function leads to the lack of time for doing responsibilities such as marital role or caring about husbands. Women considered this issue as amoral one. In this regard, findings of a qualitative study emphasized that other roles added can affect different aspects of health (Parvizy, Kiani, & Ivbijaro, 2013; (Parvizy, Naseri, Seyed Fatemi, & Ghasem Zadehkakroudi, 2010). The results of another research also emphasized that women having multi-dimensional roles suffer from inappropriate health (Hildingh, Luepker, Baigi, & Lidell, 2006). While another study concerning women health shows the husbands’ role in women’s health is inevitable (Parvizy, Seyyed Ftemi, & Kiani, 2008). These all show that if the women cannot create a balance among various responsibilities, they will face moral challenges in determining priorities. Meanwhile, the results of a research stated that women having various functions at home handled their functions to be able to perform their tasks well (Joosten & Safe, 2014).

Based on the statements of the participants, one of the family challenges women confronted with was a contradictory feeling regarding some behaviors their children had. Being sick of the children instead of helping is among those sentimental issues. These sentimental behaviors are considered as a moral challenge for women’s health. The items mentioned above are consistent with the results of a qualitative study in which children’s behavior reflected women’s health (Parvizy, Seyyed Fatemi, & Kiyani, 2009). In another research, Indian women also confirmed that their children’s misbehavior affected their health and did not know how to react against children’s behaviors (Travasso, Rajaraman, & Heymann, 2014).

Viewing moral issues that women were faithful to is among individual challenges women faced. Being impatient over time and gradual change in women’s tolerance are a few examples to name. However, treating in this way can have no satisfactory consequences for them. In this regard, the results of a study in India highlighted that because of life problems women think about committing suicide, these thoughts were not compatible with their moral issues and they are not satisfied to have such thoughts (Travasso, Rajaraman, & Heymann, 2014). It is certain that such behavioral changes which are in contrast with women’s moral criteria can affect other aspects of their health. Based on the findings, making women accept present situation is another individual moral challenge. In some circumstances, women try to prepare a better situation, but unlike the propensity they are forced to accept the present situation because of doubts in decision-making. One of the moral issues women are confronted with his decision about remarrying. In this regard, findings of a number of studies also revealed that women’s decision-making depends on their cultural factors (Parvizy, Seyyed Fatemi, & Kiyani, 2009; Travasso, Rajaraman, & Heymann, 2014). Whenever cultural norms are not consistent with their decisions, women have no way out but being silent (Leipert & Reutter, 2005). In this regard, the results of other researches in the area, presented whenever women were forced to accept the situations, they did not feel healthy. Whenever they were morally supported by others in making decisions, they cared more about their health (Alyaemni, Theobald, Faragher, Jehan, & Tolhurst, 2013; Sharifi, Anoosheh, Foroughan, & Kazemnejad, 2014).

As we noticed, moral health concept has been studied in various cultures, and various results were resulted. In some cases, they were consistent with our society’s cultural context, but in others they were not. Therefore, the researcher recommends more qualitative studies be done in this regard because the moral health concept in middle-aged women is of high importance, and there are few qualitative studies to clarify this issue.

## 5. Conclusion

This study focused on some moral issues in middle-aged women and showed their perception about moral health can affect their individual, familial and social aspects. Although there are different interpretations about some concepts, the findings showed middle-aged women’s moral health depends on concepts such as devotion, preserving morality and moral challenges. Factors such as prioritizing the health of other members of the family, trying to save marriage, respecting values, consolidating beliefs over time, and family and individual challenges play an important role in preserving women’s moral health. On the other hand, since this research is the first qualitative study about middle-aged women’s moral health in Iran’s cultural context, its result can unveil some aspects of moral health in middle-aged women.
